# Inter-laboratory comparison of a digital multimeter measurement in Turkey

**DOI:** 10.1038/s41598-023-46617-8

**Published:** 2023-11-08

**Authors:** M. Patan Alper

**Affiliations:** Physics Department, Yeditepe University, İstanbul, Turkey

**Keywords:** Electrical and electronic engineering, Applied physics

## Abstract

The standard ISO/IEC 17043:2023 specifies the general requirements for proficiency testing providers and covers the development, operation, and reporting of proficiency testing schemes. The standard aims to ensure that the measurement results obtained by different laboratories are comparable and traceable to international measurement standards. Inter-laboratory comparisons are still rare in the meteorological services network of calibration laboratories, and there are not enough bodies accredited with the ISO/IEC 17043:2023 standard in Turkey. The existing inter-laboratory comparison measurements do not fully comply with the requirements of ISO/IEC 17043:2023 and are not compatible with the measurement capabilities and scopes of the laboratories, leading to difficulties when comparing meteorological data from different laboratories. This article presents a case study of a digital multimeter interlaboratory comparison measurement in accordance with the ISO/IEC 17043:2023 standard. The results obtained were analysed using statistical methods (*En* score) and compared to the reference values provided by a secondary accredited calibration laboratory. The analysis showed that the measurement results obtained by the participants were consistent and generally within the acceptable range of *En*. The case study presented in this paper demonstrates the effectiveness of the standard in ensuring reliable measurement results in a practical setting.

## Introduction

Digital multimeters (DMMs) are essential tools used in various industries for measuring electrical parameters such as voltage, current, and resistance. Therefore, it is crucial to ensure that DMMs provide accurate and reliable measurements. Accredited calibration laboratories play a critical role in this process, as they are responsible for calibrating DMMs to ensure that they meet the required accuracy and traceability standards.

One important aspect of ensuring the accuracy and quality of DMM measurements is inter-laboratory comparison (ILC) measurements. ILC involves comparing measurement results obtained by different laboratories using the same test items and can help identify any differences in measurement practices or equipment. ILC measurements are a key element of the ISO/IEC 17043:2023 standard^1^, which provides guidelines for conducting ILC measurements of various types of measurement equipment, including DMMs.

This paper aims to provide a scientific and professional discussion of ILC measurements of DMMs in accredited calibration laboratories in accordance with the ISO/IEC 17043:2023 standard. The paper will outline the key elements of the standard, statistical design, determination of assigned values, production of PT item, the analysis of data, evaluation of performance and the reporting of results. The paper will also describe a case study of ILC measurement of DMM conducted by high level secondary electrical accredited laboratory and highlight the benefits of participating in ILC programs for accredited calibration laboratories.

Laboratories are required to participate in interlaboratory comparison measurement and/or proficiency tests in accordance with Article 7.7.2 of the ISO/IEC 17025 standard^2^. This allows laboratories to maintain their accreditation and provide accurate results. To achieve successful results, it is essential that laboratories participate in ILCs, or PTs organized according to the ISO/IEC 17043:2023 standard. The current study is a candidate for proficiency testing as it fulfils all the requirements of the standard at the accreditation application stage^3,4^.

Before the benchmark measurement, DMM is calibrated and traceable by an accredited secondary level calibration laboratory. In accordance with the guidelines set forth by ISO/IEC 17043:2023, it is imperative to justify and meticulously document the procedure for determining the assigned values within a proficiency-testing scheme. The robustness of this procedure hinges on factors such as metrological traceability and measurement uncertainty, both of which substantiate the appropriateness and effectiveness of the proficiency-testing scheme in achieving its intended objectives.

To attain the reference values for our proficiency-testing scheme, we undertook a meticulous selection process that culminated in the collaboration with an esteemed and accredited calibration laboratory. This laboratory boasts a wealth of experience and expertise in the realm of electrical calibrations—A critical aspect of our proficiency-testing scheme. Their history of providing reliable and accurate reference values for similar endeavours is a testament to their competence and proficiency.

Importantly, the chosen reference laboratory has participated in a prior inter-laboratory comparison orchestrated by the national metrology institute. The successful outcomes of this comparison served as a validation of their capabilities and underscored their ability to consistently deliver accurate results. The participation of the reference laboratory in such initiatives demonstrates their commitment to adhering to the highest standards of measurement accuracy and traceability.

The selection of the reference laboratory was not arbitrary; rather, it was a result of careful consideration. We ensured that the laboratory’s measurement capabilities and uncertainties exceeded those of the participating laboratories. This strategic choice bolstered the reliability and accuracy of the assigned reference values, contributing to the overall credibility of our proficiency-testing scheme.

Care has been taken to ensure that the uncertainty values of the participants do not exceed those of the assigned value, which is the reference value determined through calibration by an accredited calibration laboratory. In Turkey, a highly proficient and experienced pilot laboratory has been utilized for electrical calibrations, surpassing other laboratories in quality.

It is very important that the participating laboratory participates in the measurements if they are accredited, using the method for which they are accredited. Calculation of uncertainty of measurement is done using Guidance on Expressing Uncertainty in Measurement (GUM)^5^, ILAC P14:09/2020 ILAC Policy for Measurement Uncertainty in Calibration^6^ and EA-4/02 Evaluation of Measurement Uncertainty in calibration methods^7^. The ISO 13528:2022 standard is taken into account in the comparison of the results of all laboratories with the reference value and in the scoring process^8–10^.

Moreover, as mandated by ISO/IEC 17043:2023, we proactively addressed potential risks in the proficiency-testing comparison process. A thorough preliminary assessment was conducted to identify and evaluate possible risks, resulting in the implementation of appropriate measures.

One risk identified was the potential for significant deviations in readings from devices currently in use. To mitigate this, a contingency plan was established, featuring a readily available backup device for substitution. Additionally, confidentiality concerns were addressed by having participants sign agreements to uphold data privacy.

To ensure fairness and impartiality, measures were taken to prevent the sharing of results among participants who might recognize each other’s outcomes.

## Method

The Yeditepe University Physics Department’s Interlaboratory Comparison Measurement Unit, which has been accredited to the ISO/IEC 17025 standard and is in the process of successfully completing the accreditation process for the ISO/IEC 17043 standard, was responsible for organizing and conducting the ILC measurement. The ILC measurement of DMMs was conducted in accordance with the ISO/IEC 17043 standard. Measurements were performed using a stable and traceable DMM with the participation of three accredited calibration laboratories. Participants were expected to use the experimental method, calibration or measurement procedure of their choice that was appropriate to their routine use.

Prior to commencing the measurements, participants are required to acknowledge and implement the instructions outlined in the protocol that is provided along with the device. This protocol contains pertinent details regarding the device itself, the requisite settings that must be established prior to use, and participants must also confirm that the DMM has not suffered any damage during the transportation process^11–14^.

The following outlines the method used when planning the ILC measurement:

### Selection of participants

When planning the ILC, accredited calibration laboratories that can be participants are first invited to the measurements with an invitation letter. In the invitation letter, detailed information about the measurements is given (device information, quantity to be measured, traceability, measurement range, measurement uncertainty, etc.). Thanks to this information, the participant decides whether the ILC is suitable for his or her measurement capability and scope.

### Selection of the appropriate ILC test item

Stability and homogeneity of the selected reference device is one of the prerequisites for interlaboratory comparison measurement and/or proficiency tests. Homogeneity is not applicable in electronic devices, rather it is a criterion that must be met for samples. For this reason, the stability tests of the circulating device were tested before the measurements, and it was decided that the Keysight 34470A 7 1/2 Digit Multimeter in the image below was suitable for measurements. The conditions determined for the measurements: for the DMM to reach thermal equilibrium, it should be kept in operation for 24 h, preferably at (23 ± 1) °C and (45 ± 10) %Rh relative humidity. Detailed manufacturer's specifications of the ILC test item are given in Table 1^15^.

**Table 1:** Reference DMM information used in ILC measurement.

Reference DMM	
Manufacturer	KEYSIGHT
Model	34470A 7 1/2 digit multimeter
Serial number	MY57701749
Power supply	AC mains type, 220 V/50 Hz


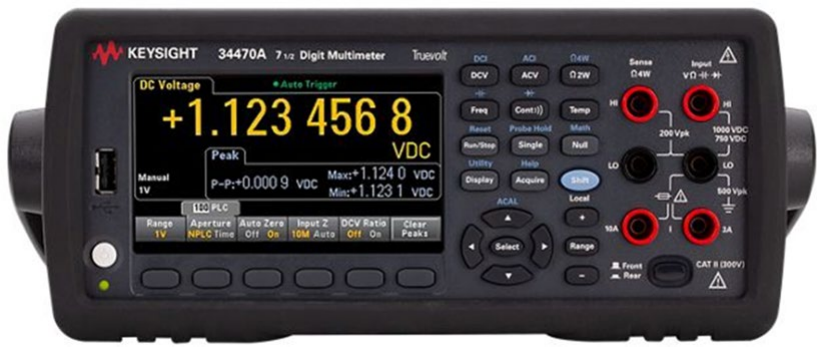


### Design of ILC protocol

A fully planned ILC protocol in accordance with the ISO/IEC 17043 standard is key in the measurement process and evaluation. The protocol designed for DMM was created considering the following topics: Scope of ILC, Participants and Confidentiality, Technical information on the ILC Device, Documents Used During ILC Measurements, Obtaining Reference Value, Basis for Measurement, Measurement Method, Measurement Points, Reporting Results, Measurement Results and Evaluation of Results.

### Determination of assigned value and stability measurements

The calibration and stability tests for the DMM used in the proficiency test were conducted in the same calibration laboratory, which has been accredited under the ISO/IEC 17025 standard as a secondary level electrical calibration laboratory. To determine the stability of the DMM, measurements were taken at identical measurement points with a two-week interval, and the disparity between the readings was found to be within the range of measurement uncertainty. The DMM was subsequently calibrated within the measurement range specified in Table 2. Due to limited participation from laboratories, stability testing was not carried out between measurements. Following the completion of the measurements by all participants, the device underwent recalibration, and the resulting measurements were evaluated. Even though there was no statistically significant difference between the pre- and post-test readings, the uncertainty was taken into consideration during the calculations.

**Table 2:** ILC measurement points.

Quantity	Measurement points	Frequency range
DC voltage	100 mV–1000 V	
AC voltage	100 mV–750 V	55 Hz, 1 kHz, 50 kHz
DC current	1 mA, 10A	
AC current	100 µA, 10A	50 Hz, 1 kHz, 5 kHz
Resistance (4W)	1 kΩ, 10 MΩ	
Resistance (2W)	10 MΩ,100 MΩ	

### Analysis of data

Before starting measurements, the participants were informed in the protocol about which measurement points and under which conditions they would measure. To make a standard evaluation, all participants were asked to present the measurement results at nominal values. The measurement results obtained by different laboratories were compiled and analysed using statistical methods to determine the degree of agreement between the results. The analysis involved calculating the mean, standard deviation, and uncertainty of the measurements. However, the ILC organizer did not provide participating laboratories with a method for calculating measurement results and measurement uncertainty. It was important for the laboratories to participate in the ILC with the method they were accredited to see their own capabilities.

### Reporting of results

A report was prepared detailing the measurement results obtained by each laboratory, along with a comparison with the reference values provided by the accredited calibration laboratory. The report also included the measurement uncertainties and statistical analysis of the data. During the evaluation of the results, special codes were given to the laboratories to ensure confidentiality and their names were not published in the report.

### Assessment of measurement capability

The measurement capability of each participating laboratory was assessed based on the degree of agreement between their measurement results and the reference values. The assessment included an evaluation of the measurement uncertainty and the laboratory's compliance with the ISO/IEC 17043 standard.

Overall, the method used for conducting the ILC measurement of DMMs in accordance with the ISO/IEC 17043 standard involved careful selection of test items, standardization of measurement procedures, statistical analysis of data, and detailed reporting of results^16–18^. The method helped to ensure the accuracy, comparability, and traceability of measurement results and provided a valuable assessment of the measurement capability of the participating laboratories.

### Ethical approval

I would like to clarify that no human participants were involved in this study. All data were obtained in a laboratory setting, and no data collection or analysis involving human subjects took place. Consequently, there were no ethical parameters, ethics approval, or informed consent issues related to human participants in this research.

## Measurements and results

The ILC measurement of digital multimeters (DMMs) conducted by multiple accredited calibration laboratories using different types of DMMs yielded consistent and reliable measurement results in accordance with the ISO/IEC 17043 standard.

The measurement results obtained from each laboratory were compared against the reference values provided by an accredited calibration laboratory. The assessment of these results was conducted following the statistical techniques outlined in the ISO/IEC 17043 and ISO 13528 standards. Although various methods for evaluation exist within the standard (such as zet, zeta, and *En*), the *En* evaluation method was selected for this PT measurement.

The adoption of the En score evaluation method in our Interlaboratory Comparison (ILC) study was driven by the need to ensure the traceability and reliability of our measurements. By utilizing this approach, we enhance the credibility of our findings, as they are rooted in the precision and traceability offered by an accredited calibration laboratory. Furthermore, it is essential to note that we followed Scenario 1 of EA 4/21, wherein the organizer utilized an assigned value based on an external reference^19^. This adherence to international standards further strengthens the robustness and trustworthiness of our ILC results.

Our decision to utilize the *En* score evaluation method aligns with the guidance provided by the ISO 13528 standard, specifically recommended for proficiency testing among calibration laboratories. By employing this method, we aim to maintain consistency with established best practices within the field. The *En* score evaluation method was chosen primarily due to its capability to uphold the traceability of the PT device. This ensures that our measurements are firmly anchored to a precisely defined reference value. The performance evaluation method used in ILC measurements is made according to the equation given below in the form of *En* score calculation.

1 $${E}_{n}=\frac{{X}_{lab}-{X}_{ref}}{\sqrt{{U}_{lab}^{2}-{U}_{ref}^{2}}},$$

*X*_*lab*_ is the participant’s result, *X*_*ref*_ is the assigned value, *U*_*lab*_ is the expanded uncertainty of a participant's result, *U*_*ref*_ is the expanded uncertainty of the reference laboratory's assigned value.

If |*En*|≤ 1, the measurement result is considered as satisfactory. If |*En*|> 1, the measurement result is considered as unsatisfactory.

The comparison of measurement results also highlighted the importance of following standardized methods for conducting ILC measurements of DMMs. The use of standardized measurement procedures, careful selection of test items, and statistical analysis of data helped to ensure the accuracy, comparability, and traceability of measurement results across different laboratories.

Accurate calculation of measurement uncertainty is a critically important aspect of ILC measurements. In this regard, both the laboratory providing the reference value and the participating accredited laboratories adhere to international standards when selecting and applying appropriate methods for uncertainty estimation. This ensures the incorporation of consistency and similar parameters in uncertainty assessments. This topic is compiled and discussed to ensure conformity in uncertainty calculations. Measurement uncertainties are classified as Type A or Type B evaluations. Type A involves statistical methods like calculating standard deviations from readings or fit residuals. Type B involves non-statistical methods like using past data or specifications. Both types become standard uncertainties. Sensitivity coefficients show their influence on the measured quantity. When uncorrelated, combined standard uncertainty (u_c_) is the root-sum-square of squared uncertainties and coefficients. Expanded uncertainty widens the range with a coverage factor (k): U = k * u_c_, based on degrees of freedom for confidence. The concepts are detailed in the Guide to the Expression of Uncertainty in Measurement (GUM)^5^.

When it comes to accurate measurements, understanding the factors that contribute to uncertainty is crucial. An uncertainty budget helps us see these parameters and estimate the overall uncertainty in a measurement. For voltage measurements, several things can affect the final result. We'll discuss a sample uncertainty budget to explain how we manage uncertainty in our Interlaboratory Comparison (ILC) measurements for ILC item. In calculating the measurement uncertainty, the accredited laboratory providing the reference value has taken into account the following parameters for 10 V measurement given Table 3. The reason behind presenting a measurement uncertainty greater than the calculated value is due to the accredited laboratory providing the reference value being unable to offer uncertainty lower than the scope mandated by their accreditation. This holds true not only for voltage measurements but also for current and resistance measurements, where similar parameters have been considered. Parameters within the uncertainty assessment can be classified into two groups. The first group comprises parameters originating from the reference value and auxiliary equipment used in the measurement process, while the second group encompasses parameters entirely stemming from the measured device itself, such as test resolution and test repeatability.

**Table 3:** Uncertainty estimation for 10 V DC measurement.

Uncertainty components	Uncertainty	Statistical distribution	Sens. coeff	Standard uncertainty
	(V)			(V)
Calibrator	1.0E-05	Normal	1	5.00E-06
Drift	4.0E-05	Rectangular	1	2.31E-05
Temperature	0.3E-05	Rectangular	1	1.73E-06
Resolution	0.1E-05	Rectangular	1	5.8E-07
Connection cable	0.5E-06	Rectangular	1	2.9E-07
Repeatability	2.7E-07	Normal	1	2.7E-07
			Total Unc. 2,4E-05	
			Expanded Unc. (k = 2) 4,8E-05	

Table 4 presents the *En* values for every measurement point and for all three participating laboratories with reference values.

**Table 4:** Summary of the measurement results, the estimated combined expanded uncertainty and En values.

Lab code	Reference		X lab			Y lab			Z lab		
Nominal value	Measured value	Expanded uncert	Measured value	Expanded uncert	En	Measured value	Expanded uncert	En	Measured value	Expanded uncert	En
DC voltage											
100 mV	100.00049	1.60E-03	100.00010	3.0E-03	0.1	99.999900	6.0E-03	0.1	100.000002	6.0E-02	0.0
1 V	1.0000043	7.90E-06	0.9999997	1.7E-05	0.2	1.0000014	9.0E-06	0.3	1.0000153	3.0E-04	0.0
10 V	10.000008	7.40E-05	9.9999910	1.6E-04	0.1	10.000016	1.4E-04	0.0	10.0001050	2.0E-03	0.0
100 V	100.00108	7.40E-04	100.00044	2.0E-03	0.2	100.000860	8.4E-04	0.2	100.000410	1.0E-02	0.1
1000 V	1000.0034	7.40E-03	999.99590	2.2E-02	0.3	1000.00780	8.2E-03	0.3	1000.02710	6.0E-01	0.0
AC voltage											
100 mV 55 Hz	99.995400	1.4E-02	99.997000	2.2E-02	0.1	99.962000	3.6E-02	0.9	99.977000	1.8E-01	0.1
100 mV 1 kHz	99.998200	1.4E-02	100.00000	2.2E-02	0.1	99.964000	3.7E-02	0.8	99.970000	1.8E-01	0.2
100 mV 50 kHz	100.06790	3.9E-02	100.078000	3.6E-02	0.3	99.995000	5.3E-01	0.1	–	–	–
1 V 55 Hz	1.0000070	1.1E-04	0.999968	2.0E-04	0.2	0.9999550	1.4E-04	0.3	0.9998010	2.0E-03	0.1
1 V 1 kHz	0.9999970	1.1E-04	0.9999900	2.0E-04	0.0	0.9999890	1.2E-04	0.0	0.9998260	2.0E-03	0.1
1 V 50 kHz	1.0006220	3.4E-04	1.0003200	2.9E-04	0.5	1.0002790	2.3E-03	0.1	1.0004080	2.0E-03	0.1
10 V 55 Hz	9.9995500	1.3E-03	9.9995200	2.5E-03	0.0	9.9997200	1.3E-03	0.1	9.9979700	2.0E-02	0.1
10 V 50 kHz	10.005580	1.6E-03	10.006000	3.5E-03	0.1	10.002720	9.0E-02	0.0	10.004800	2.0E-02	0.0
100 V 55 Hz	99.993100	1.1E-02	99.992000	2.5E-02	0.0	100.00030	2.6E-02	0.2	99.983100	2.0E-01	0.0
100 V 1 kHz	99.994500	8.4E-03	100.00800	2.5E-02	0.5	100.00300	2.6E-02	0.3	99.985300	2.0E-01	0.0
100 V 50 kHz	100.03780	1.6E-02	99.947000	2.0E-01	0.5	100.01550	5.3E-01	0.0	–	–	–
750 V 55 Hz	749.95700	1.5E-01	749.86200	2.0E-01	0.4	749.96580	3.9E-01	0.0	749.96700	1.0E + 00	0.0
750 V 1 kHz	749.95500	1.5E-01	749.97000	2.0E-01	0.1	749.95010	3.9E-01	0.0	749.99900	1.0E + 00	0.0
DC current											
+ 1 mA	0.9999782	1.9E-05	1.0000069	1.3E-04	0.2	0.9999737	8.3E-05	0.1	1.0000301	7.0E-04	0.1
+ 10 mA	10.001175	2.1E-04	10.001299	1.3E-03	0.1	10.000570	6.8E-04	0.9	10.0012479	7.0E-03	0.0
+ 100 mA	100.01167	3.0E-03	100.00307	1.1E-02	0.6	100.00324	8.0E-03	0.8	100.015913	6.0E-02	0.1
+ 1 A	1.0000598	6.4E-05	0.9999942	3.3E-04	0.3	0.9996929	1.5E-04	1.5	1.0000157	6.0E-04	0.2
+ 2 A	1.9999489	1.8E-04	–	–	–	1.9995461	1.9E-03	0.1	–	–	–
+ 5 A	4.9999060	3.9E-04	4.9992530	5.5E-03	0.1	5.000008	4.8E-03	0.0	4.9991937	4.0E-03	0.2
+ 10 A	10.008064	7.4E-04	10.104215	9.4E-03	10.2	10.003527	7.2E-03	0.6	–	–	–
AC current											
100 μA 50 Hz	100.0136000	4.8E-02	99.954000	2.1E-01	0.3	99.970000	3.1E-01	0.1	99.986000	7.0E-01	0.0
100 μA 1 kHz	100.0137000	4.8E-02	99.987000	1.1E-01	0.2	99.947000	2.7E-01	0.2	99.973000	7.0E-01	0.3
100 μA 5 kHz	99.9978000	4.8E-02	100.20700	3.5E-01	0.6	100.38900	7.6E-01	0.5	99.966000	1.1E + 00	0.0
1 mA 50 Hz	0.9998910	2.2E-04	0.9999760	1.0E-03	0.1	0.9999200	2.4E-02	0.0	0.9997970	3.0E-03	0.0
1 mA 1 kHz	0.9999690	2.1E-04	0.9999900	2.1E-03	0.0	0.9998900	2.7E-02	0.0	0.9998810	3.0E-03	0.0
1 mA 5 kHz	0.9999230	2.2E-04	1.0000500	4.1E-03	0.0	0.9998830	2.7E-02	0.0	0.9998240	3.0E-03	0.0
1 A 50 Hz	1.0000890	2.2E-04	1.0001350	5.5E-04	0.0	1.0003380	1.3E-03	0.1	0.9997040	2.0E-03	0.2
1 A 1 kHz	1.0001600	2.2E-04	0.9999300	6.0E-03	0.1	1.0003630	1.5E-03	0.1	0.9998300	2.0E-03	0.2
2 A 50 Hz	2.0005040	6.3E-04	1.9999610	3.1E-03	0.2	2.0001680	5.1E-03	0.1	1.9989240	4.0E-03	0.4
2 A 1 kHz	2.0001790	6.3E-04	2.0002900	1.1E-03	0.1	1.9992700	5.2E-03	0.2	1.9990040	4.0E-03	0.3
2 A 5 kHz	2.0007780	1.7E-03	–	–	–	1.9975930	5.5E-03	0.6	–	–	–
10 A 50 Hz	10.0089500	8.1E-03	10.106810	7.0E-03	9.1	10.011630	2.9E-02	0.1	–	–	–
10 A 5 kHz	10.0190100	8.1E-03	10.102100	7.0E-03	6.5	9.9864200	2.9E-02	1.1	–	–	–
Resistance (2W)											
10 MΩ	9.9999670	3.4E-04	10.000137	3.5E-03	0.0	9.999658	7.2E-03	0.0	9.99943	8.0E-05	1.5
100 MΩ	99.9936000	7.9E-03	100.04195	4.7E-02	1.0	99.89587	7.4E-01	0.1	–	–	–
Resistance (4W)											
100 Ω	100.0013400	7.7E-04	99.99810	1.5E-02	0.2	100.0011	2.0E-03	0.0	100.0122	6.0E-02	0.2
1 kΩ	1.0000285	6.6E-06	1.0000089	4.1E-05	0.2	1.0000307	3.9E-05	0.2	1.000064	1.0E-06	1.3
10 kΩ	10.0002980	7.6E-05	10.000072	4.0E-04	0.2	9.999768	2.2E-04	0.8	10.000848	1.0E-05	1.5
100 kΩ	100.0023900	9.1E-04	100.00148	3.8E-03	0.1	100.00007	1.5E-03	0.7	100.00275	1.0E-04	0.4

**Figure 1:**
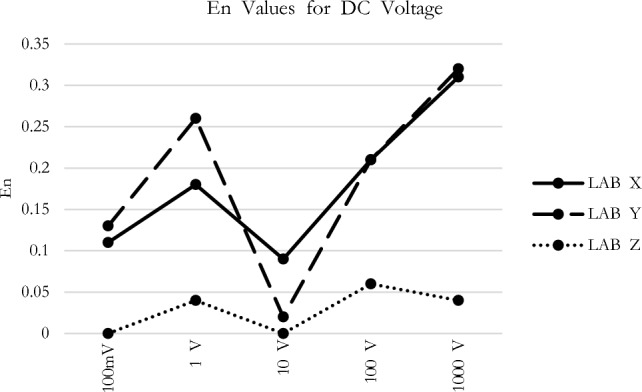
*En* values for DC voltage.

**Figure 2:**
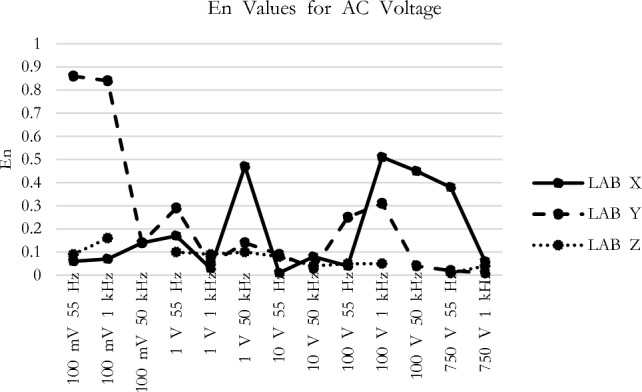
*En* values for AC voltage.

**Figure 3:**
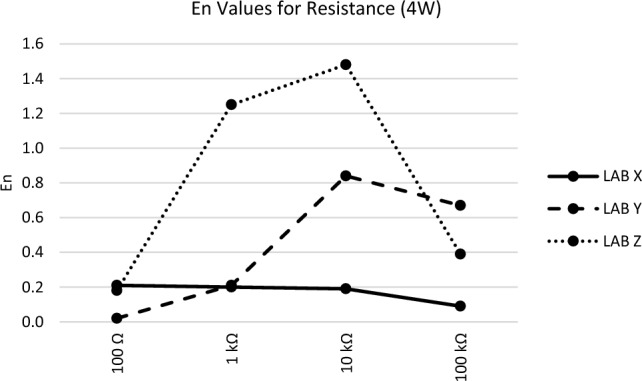
*En* values for DC current.

**Figure 4:**
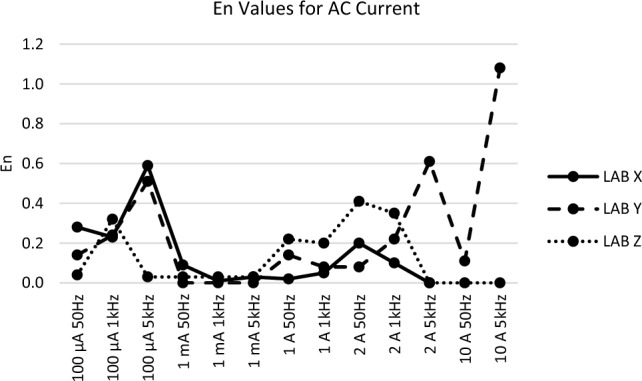
*En* values for AC current.

**Figure 5:**
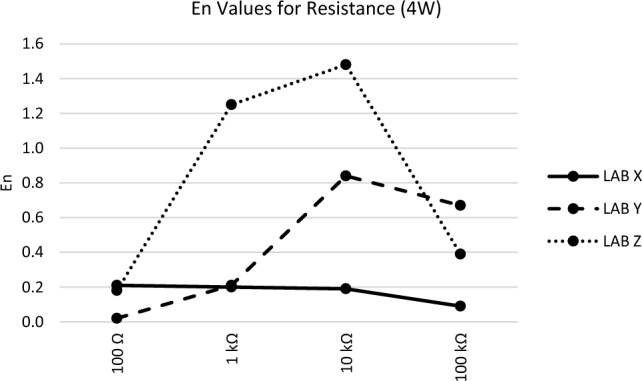
*En* values for resistance (4W).

The *En* values observed at each measurement point for all participants during the comparison measurement presented in Figs. 1, 2, 3, 4 and 5 are consistent with the findings reported in Table 3.

In Fig. 1, the DC Voltage *En* scores for all participants have remained below a certain threshold. When examining both low and high voltage results, it can be stated that the participants have achieved successful measurement outcomes. Figure 2, while the AC voltage *En* scores generally indicate favorable results, there is a notable observation in the case of Lab Y’s 100 mV measurement. Although the *En* value does not exceed a critical level, it might be appropriate for the participant to conduct a preventive activity analysis by investigating the rationale behind the 0.9 value.

For the DC and AC current plots in Figs. 3 and 4, Lab Y exhibits *En* values surpassing unity at two points. What draws attention during the results analysis is the substantial deviation obtained by the participating laboratory while measuring using the circulating instrument. Given the deviation of the reference device employed by the participant, an assessment could be made concerning its impact on traceability conditions.

Figure 5, in the resistance measurement results, Lab X and Y achieve successful outcomes, while Lab Z encounters failures at two points. Notably, during the analysis of measurement results, it is evident that the participant laboratory offers low uncertainty at these particular points. Consequently, revisiting the parameters related to measurement uncertainty would serve as a corrective action initiation.

These additional observations and analyses enhance the discussion around the obtained results, shedding light on specific instances where participant laboratories demonstrated strong performance and identifying areas where corrective measures could contribute to further enhancing the study’s reliability and overall quality.

The ILC measurement also provided a valuable assessment of the measuring capacity of the participating laboratories. The evaluation revealed that most laboratories were able to obtain accurate and reliable measurement results and measurement uncertainties were within acceptable limits. The assessment also helped identify areas for improvement and further development of measurement procedures and equipment (Supplementary Information).

## Conclusion

This study conducted an inter-laboratory comparison measurement through collaborative efforts, with results evaluated according to the ISO/IEC 17043 standard. By adhering to this standardized approach, we attained an objective assessment of participants' performance. Among 122 measurements across 3 laboratories, 8 values exhibited |En|> 1. The occurrence of En values surpassing 1 for specific participants can be attributed to a blend of factors, including variations in laboratory setups, equipment limitations, and calibration inconsistencies. Collectively, these factors contribute to heightened measurement uncertainty. Moreover, certain complex measurements, particularly in cases such as Lab Y’s AC voltage and current results, may lead to minor deviations. These insights emphasize the necessity of thorough uncertainty analysis and strict adherence to standardized protocols, ensuring consistent and accurate inter-laboratory comparisons. Within this ISO/IEC 17043-guided comparative assessment, certain aspects of laboratory performance proved unsatisfactory. Remedial measures, beyond the established criteria, will be necessary. Consequently, recommendations emerge from these evaluations to mitigate En values. One approach involves heightened attention to uncertainty calculation, employment of improved equipment for enhanced measurement accuracy, and verification of reference device traceability. Implementation of these suggestions can enhance laboratory performance, ultimately yielding more dependable and precise outcomes. Overall, these results underscore the feasibility of objectively measuring and enhancing laboratory performance.

### Supplementary Information

Supplementary Information.

## Data Availability

In this study, the data were not obtained from any specific database; rather, the measurement results were directly derived from the participants involved in the study. Therefore, there are no database links, references, or access numbers to provide. The measurements were conducted as part of the study procedures, and the results were obtained directly from the participants' responses. The raw data sets generated and/or analysed during the current study are available from the corresponding author (M.P.A) on reasonable request. All methods were conducted in compliance with the relevant rules and regulations.
